# A new lineage of braconid wasps in Burmese Cenomanian amber (Hymenoptera, Braconidae)

**DOI:** 10.3897/zookeys.730.22585

**Published:** 2018-01-17

**Authors:** Michael S. Engel, Diying Huang, Chenyang Cai, Abdulaziz S. Alqarni

**Affiliations:** 1 Division of Entomology, Natural History Museum, University of Kansas, 1501 Crestline Drive – Suite 140, Lawrence, Kansas 66045-4415, USA; 2 Department of Ecology & Evolutionary Biology, University of Kansas, Lawrence, Kansas 66045, USA; 3 Division of Invertebrate Zoology, American Museum of Natural History, Central Park West at 79th Street, New York, New York 10024-5192, USA; 4 State Key Laboratory of Palaeobiology and Stratigraphy, Nanjing Institute of Geology and Palaeontology, Chinese Academy of Sciences, Nanjing 210008, People’s Republic of China; 5 Department of Plant Protection, College of Food and Agriculture Sciences, King Saud University, P.O. Box 2460, Riyadh 11451, Kingdom of Saudi Arabia

**Keywords:** Cretaceous, Euhymenoptera, fossil, Ichneumonoidea, Myanmar, parasitoid, taxonomy, wasp

## Abstract

A new braconid wasp from the Upper Cretaceous (Cenomanian) amber of the Hukawng Valley in Kachin State, Myanmar is described and figured from a unique female. *Seneciobracon novalatus* Engel & Huang, **gen. et sp. n.**, is placed in a distinct subfamily, Seneciobraconinae Engel & Huang, **subfam. n.**, owing to the presence of a unique combination of primitive protorhyssaline-like traits, with an otherwise more derived wing venation. The fossil is discussed in the context of other Cretaceous Braconidae.

## Introduction

Although braconids are a frequently encountered lineage in the modern hymenopteran fauna (Grimaldi and Engel 2005; Quicke 2015), and are represented in Cenozoic ambers by diverse subfamilies (e.g., Brues 1933, 1939; van Achterberg 1982, 2001; Tobias 1987; Zuparko and Poinar 1997; Engel and Bennett 2008; Butcher et al. 2014), their presence in fossiliferous resins from the Cretaceous is comparatively scant. This diverse clade of parasitoid wasps is today represented by over 21,200 described species (Aguiar et al. 2013; Yu et al. 2016), and with an actual diversity of perhaps more than double what is already known (Quicke 2015). Based on current estimates of relationships among the subfamilies (Sharanowski et al. 2011) and coupled with the breadth of these same groups represented in Lower Cenozoic deposits, it is clear that much of the cladogenesis among the higher groups within the family had already transpired, with these lineages extending well into the Cretaceous. It is therefore surprising that so few braconids have been recovered from Cretaceous deposits, and although several interesting new taxa have been described recently, they remain relatively rare with hitherto only 11 formally named species (*vide* Discussion, *infra*). Nonetheless, those that have been documented are interestingly phylogenetically basal to crown-group Braconidae (e.g., Perrichot et al. 2009), and so have the greatest potential for illuminating our understanding of the phases of braconid diversification.

**Figure 1.**
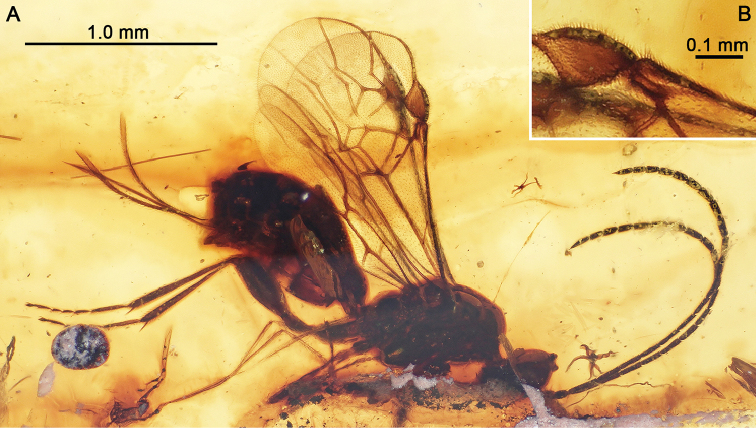
Photographs of holotype female (NIGP 164784) of *Seneciobracon novalatus* Engel & Huang, gen. et sp. n., in mid-Cretaceous amber from northern Myanmar. **A** Holotype in right lateral view as preserved **B** Inset detail of pterostigmal region of forewing, depicting small costal cell.

Here we describe a new genus and species of braconid wasps in Burmese amber (Fig. 1A). The genus is interesting in that it intermixes primitive and derived features in a unique combination not attributable to any of the recognized subfamilies, and is therefore placed within a new, extinct subfamilial lineage putatively more closely related to modern cyclostome braconids than the basal Eoichneumoninae and Protorhyssalinae. The subfamily is similar to modern Rhyssalini, sharing varied plesiomorphies with this group, but can be distinguished in features of the notal and metasomal structure as well as wing venation and putatively apomorphic effacement of the occipital carina.

## Material and methods

A small flake of Upper Cretaceous amber from Myanmar was discovered to contain a tiny braconid wasp, which is here designated as the holotype for the species described. The chip of amber is 8.9 mm at its maximum length, 5.4 mm in maximum width, and approximately 1.7 mm deep. While flat surfaces could be polished on the larger planes, permitting lateral views of the specimen, the narrow edges are rough and could not be cleaned further owing to the close proximity of the wasp’s anterior end near one border (Fig. 1A). Overall, however, the wasp is in exceptional condition, with the antennae extended upward and curving back toward the body, the legs either extended or folded beneath the body, and the ovipositor extended. The wings extend above the body and although their apical quarters are slightly bent (each wing bending slightly to the animal’s left, and therefore into the background in figure 1A), the venation can be seen beautifully. The only challenging details to discern are those of the metasoma, where much is hidden by small lateral fractures along the body, and a darkening of the amber near the body. The same holds for portions of the mesosoma, but is not as impactful on observations for this tagma.

We document the present fossil in the interest of elaborating character combinations of Cretaceous Braconidae and in the hopes that these will ultimately aid our resolution of basal relationships among lineages of braconids, with descriptive work such as this forming the basis for such discovery (sensu Grimaldi and Engel 2007). For the morphological account we have used an amalgamation of the morphological terminologies proposed by Huber and Sharkey (1993), van Achterberg (1993), and Sharkey and Wharton (1997), the latter two specific to Braconidae. Photographs of the holotype were taken through an Infinity K-2 long-distance microscope lens, using a Canon 7D digital camera, while line drawings were made with the aid of camera lucida attached to an Olympus SZX-12 stereomicroscope. The specimen was measured using the same stereomicroscope and done with the aid of an ocular micrometer. The amber locality has been mapped by Cruickshank and Ko (2003) and Grimaldi and Ross (2017), who also provide a geological account of the deposits. The amber has been dated to the earliest Cenomanian (approximately 98.8 Ma) (Shi et al. 2012).

## Systematic paleontology

### Family Braconidae Nees von Esenbeck

#### Seneciobraconinae

Taxon classificationAnimaliaHymenopteraBraconidae

Engel & Huang subfam. n.

http://zoobank.org/152DB385-88D6-441C-B480-9D824396FF8D

##### Type genus.

*Seneciobracon* Engel and Huang, gen. n.

##### Diagnosis.

**Figure 2.**
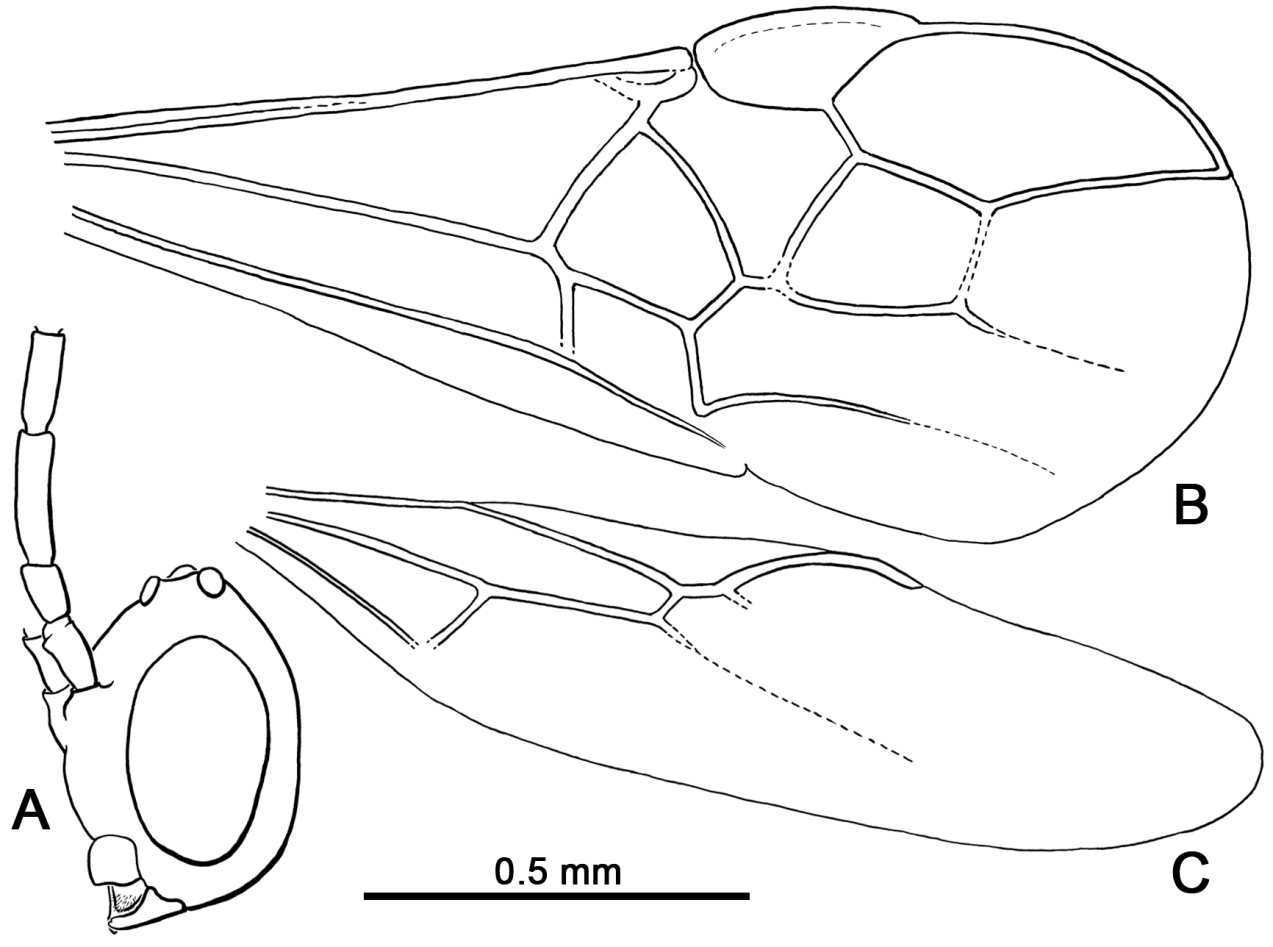
Head and wing venation of *Seneciobracon novalatus* Engel & Huang, gen. et sp. n., in Burmese amber. **A** Head in left lateral view **B** Forewing. **C** Hind wing.

Head orthognathous, cyclostome; clypeus shorter than wide, protruding; hypoclypeal depression deep (Fig. 2A); mandibles short, about as long as compound eye width; antenna filiform, with 19 flagellomeres; flagellum with sparse multiporous plate sensilla; occipital carina present but incomplete, present and strong only near mandible, otherwise effaced; compound eyes without ocular setae. Pronotal collar distinct; notauli deeply impressed, percurrent, simple, not meeting posteromedially; mesoscutal lateral areas swollen, smooth; mesoscutellum slightly raised relative to surface of mesoscutum; epicnemial carina present; postpectal carina absent; precoxal sulcus absent. Forewing (Figs 1A, 2B) with short, narrow costal cell, otherwise C+Sc+R fused along length; 1Rs exceedingly short, forming straight line with 1M; rs-m present, nebulous (i.e., two closed submarginal cells); 1m-cu meeting first submarginal cell (thus Rs+M divided into long 1Rs+M and short 2Rs+M); 2m-cu absent; 1cu-a postfurcal; 2cu-a absent; stubs of 1a and 2a absent. Hind wing (Fig. 2C) with sc+r-m lacking bulla, much shorter than 1M; m-cu absent; bulla present between 1A and apex of 1Cu; 2Cu absent. Metasomal tergum I apparently without dorsope; ovipositor elongate but slightly shorter than metasoma.

#### Seneciobracon

Taxon classificationAnimaliaHymenopteraBraconidae

Engel & Huang gen. n.

http://zoobank.org/F589489E-A107-4161-A188-A3A19151ADAA

##### Type species.

*Seneciobracon novalatus* Engel & Huang, sp. n.

##### Diagnosis.

As for the subfamily (*vide supra*).

##### Etymology.

The new generic name is a combination of the Latin *senecio*, meaning, “old man”, and *Bracon* Fabricius, type genus of the family. The gender of the name is masculine.

#### Seneciobracon novalatus

Taxon classificationAnimaliaHymenopteraBraconidae

Engel & Huang sp. n.

http://zoobank.org/4B94ADA2-48F7-4EB2-B3FE-DE4DEBD141DA

Figs 1 , 2

##### Holotype.

♀ (Fig. 1A), NIGP 164784, lowermost Cenomanian (near Albian boundary), Hukawng Valley, Kachin State, northern Myanmar; deposited in the Nanjing Institute of Geology and Palaeontology, Chinese Academy of Sciences, Nanjing, China.

##### Diagnosis.

As for the subfamily (*vide supra*).

##### Description.

♀: Total length 2.0 mm (as preserved, excluding ovipositor); forewing length 1.50 mm, hind wing length 1.35 mm; integument dark brown (Fig. 1A), lighter on mouthparts, tarsi, ovipositor, and ovipositor sheaths; wing veins brown to dark brown, membranes hyaline.

Head apparently longer than broad (direct facial view not possible, observable in frontal-oblique view: Fig. 2a), impunctate and imbricate, with sparse, minute setae on face, such setae slightly longer on clypeus; face below antennal toruli faintly convex, sloping to distinct impression along epistomal sulcus; clypeus protruding, rounded, short, medial length about one-third that of length of face from antennal toruli to epistomal sulcus; hypoclypeal depression deep and wide; mandible short, just meeting opposing mandible when closed, apparently with a single, minute, subapical tooth; maxillary palpus elongate, longer than head, with 6 palpomeres, with palpomeres II–VI longer than wide, individual palpomeres with dense, minute setae; compound eye large and glabrous, length 0.28 mm, much broader than gena, inner margin not emarginate; ocelli positioned on top of vertex; ocelli well separated, median ocellus separated from lateral ocelli by approximately twice median ocellar diameter, lateral ocelli separated from posterior of head by almost twice median ocellar diameter, ocellocular distance slightly more than twice ocellar diameter; antenna slightly shorter than body length (excluding ovipositor); scape twice as long as apical width, length 0.08 mm, width 0.04 mm, truncate apically; pedicel about 1.75 times as long as wide, about as broad as scape, length 0.07 mm; flagellum with 19 flagellomeres; basal flagellomeres elongate and of approximately equivalent widths, flagellomere I length 0.15 mm, width 0.03 mm; flagellomere II length 0.12 mm; flagellomere III length 0.12 mm; remaining flagellomeres progressively tapering in length toward apex, apical flagellomeres about 2.0–2.25 times as long as wide except apicalmost flagellomere slightly more than 3 times as long as wide; multiporous plate sensilla sparse; apicalmost flagellomeres with a short, thick, peg-like seta at apex.

Mesosoma length 0.75 mm; pronotal collar distinct; pronotal surface smooth, dorsope and laterope absent; mesoscutum smooth, raised above pronotum; notauli deeply impressed, simple, percurrent but not meeting; lateral areas of mesoscutum (lateral to notauli) distinctly raised, convex; mesoscutellar sulcus deeply impressed, simple; mesoscutellum slightly raised, convex, smooth; mesopleuron smooth; propodeum areolate. Legs slender, with numerous setae; metafemur swollen; tibial spurs short, protibial calcar slightly curved, without comb; tibiae without spines or peg-like setae; metatibia length 0.63 mm; basitarsi largest tarsomeres, but shorter than combined length of remaining tarsomeres; pretarsal claws short, simple; arolium small. Forewing (Fig. 2B) with minute costal cell present apically near base with pterostigma, otherwise C+Sc+R fused along length; pterostigma large, longer than wide, with border inside marginal cell comparatively straight, anterior border convex, bulging; marginal cell large, extending nearly to wing apex, broad, broader than pterostigmal width; 1Rs exceedingly short, forming straight line with 1M; 1Rs+M originating near prestigma; 1M straight; 1Rs+M long, slightly curved, extending strongly posteriad to meet 1m-cu; 1m-cu meeting Rs+M near longitudinal tangent of M+Cu; 2Rs+M present, exceedingly short; first submarginal cell trapezoidal, but nearly triangular owing to short 2Rs+M; second submarginal cell large, nearly square, apical border formed of nebulous rs-m; r-rs arising slightly distad pterostigmal midlength, much shorter than 3Rs, at least 2 times longer than 1Rs; 1cu-a postfurcal; 1Cu shorter than 1cu-a; 2Cu much longer than 1Cu; 2cu-a absent, thus subdiscal cell open; stubs of 1a and 2a absent. Hind wing (Fig. 2C) with margins setose and secondary ‘hamuli’ (two distinctively elongate setae on anterior margin at apex of C); 3 distal hamuli present on R; R tubular on anterior wing margin for short distance, otherwise extending as nebulous vein to near wing apex; 2Sc+R distinct, longer than sc+r-m; sc+r-m without bulla; Rs tubular near base then extending as nebulous vein; 2M tubular near base then nebulous; 1Cu shorter than 1M; 2Cu absent; bulla present between apex of A and Cu.

Metasoma length 1.0 mm; terga with integument transversely wrinkled, otherwise impunctate, with sparse, minute setae; sterna apparently smooth and impunctate; tergum I about as long as wide, terga II and III apparently longer than wide, fused; remaining terga transverse; dorsope of tergum I apparently absent; ovipositor long, straight, shorter than metasoma when exerted, length 0.80 mm; ovipositor sheaths slightly broader apically, with abundant minute setae.

♂: *Latet*.

##### Etymology.

The specific epithet is a combination of the Latin *novus*, meaning, “new”, and *alatus*, meaning, “wing”, and is a reference to the more derived wing venation relative to other Cretaceous amber Braconidae (e.g., the protorhyssalines and *Aenigmabracon* Perrichot et al.).

## Discussion

The new subfamily is most similar to the modern, putatively primitive Rhyssalinae, and the tribe Rhyssalini in particular (van Achterberg 1993; Belokobylskij 2009), and both subfamilies are rather generalized cyclostomes. *Seneciobracon novalatus* has a distinctive combination of traits not found among rhyssalines such as the absence of the stub of 2a in the forewing, complete absence of m-cu in the hind wing (even as a spectral trace), presence of a well-defined 2Rs+M (1m-cu typically confluent with 2Rs in Rhyssalini), presence of a distinct costal cell at the apex of the otherwise fused C+Sc+R, incomplete occipital carina (present only near mandibles), and absence of a dorsope on metasomal tergum I. Both Seneciobraconinae and Rhyssalinae, although cyclostome, differ from the extinct Protorhyssalinae in the absence of hind wing 2Cu, a putatively derived feature as 2Cu is present in Eoichneumoninae, Protorhyssalinae, Trachypetinae, Apozyginae, and Ichneumonidae. In addition, both lack the five-sided second submarginal cell more typical of the protorhyssalines (e.g., Basibuyuk et al. 1999; Perrichot et al. 2009; Ortega-Blanco et al. 2011; Engel 2016; Engel and Wang 2016; Engel et al. 2017), a condition that results from the meeting of 1m-cu with the second submarginal cell. In this regard, *Seneciobracon novalatus* has a venation that is more similar to modern Braconidae than to any of the other mid-Cretaceous or older braconids, and cannot be included within Protorhyssalinae or the more basal stem of Braconidae (sensu Perrichot et al. 2009). The subfamily Seneciobraconinae could be interpreted as a basal tribe in a more broadly circumscribed Rhyssalinae, although the list of differences from traditional rhyssalines listed above warrants against such inclusion at this time. Accordingly, we have preferred to recognize the former as distinct pending definitive cladistic evidence for such a clade and particularly given the challenges in defining a definitively monophyletic Rhyssalinae and the composition and circumscription of the subfamily remains somewhat in flux, albeit improving (Quicke 2015). Moreover, the putatively apomorphic dorsal effacement of the occipital carina tends to further support the subfamily. A similarly derived trait is found in *Histeromerus* Wesmael (formerly as Histeromerinae), a derived rhyssaline also lacking a complete occipital carina (e.g., Sharanowski et al. 2011), but otherwise differing from the fossil described herein (e.g., van Achterberg 1984, 1992).

Quicke (2015, p. 210) questioned whether the short, narrow costal cell present apically in some Cretaceous fossil Braconidae might be an artefact of preservation. It is clear that this is not the case, as evidenced nicely in the present fossil (Fig. 1B). The same condition is present in most of the known protorhyssalines (Perrichot et al. 2009; Engel 2016; Engel and Wang 2016; Engel et al. 2017), as well as *Aenigmabracon* (Perrichot et al. 2009). This character-state appears to be a primitive feature among Cretaceous braconids.

Aside from *S. novalatus*, there have hitherto been 11 Cretaceous amber Braconidae described – *Archephedrus stolamissus* Ortega-Blanco et al. and *Protorhyssalopsis perrichoti* Ortega-Blanco et al. in Albian Spanish amber; *Archaeorhyssalus subsolanus* Engel and *Rhetinorhyssalus morticinus* Engel in Cenomanian Burmese amber, *Protorhyssalodes arnaudi* Perrichot et al. and *Aenigmabracon capdoliensis* Perrichot et al. in Cenomanian amber from France; *Protorhyssalus goldmani* Basibuyuk et al. and *Rhetinorhyssalites emersoni* Engel et al. in Turonian amber from New Jersey; and *Diorhyssalus allani* (Brues), ‘Pygostylus’ patriarchicus Brues, and ‘Neoblacus’ facialis Brues in Campanian amber from Canada (Brues 1937; Basibuyuk et al. 1999; Perrichot et al. 2009; Ortega-Blanco et al. 2009, 2011; Engel 2016; Engel and Wang 2016; Engel et al. 2017). Given their prodigious modern diversity, this representation from the Cretaceous is disappointing. Ichneumonoidea generally are uncommon in Cretaceous amber, with comparatively few species described to date. As noted by McKellar and Engel (2012) and McKellar et al. (2013a), this dearth of material is perhaps the result of a bias toward the capture of often smaller-bodied animals in amber, while modern ichneumonoids include many groups of larger sizes, particularly among Ichneumonidae, as well as smaller wasps. However, where known, most Cretaceous ichneumonoids are on the smaller end of the size spectrum, including those preserved as compressions (e.g., Townes 1973; Zhang and Rasnitsyn 2003; Kopylov 2011, 2012; Belokobylskij 2012), and one might speculate that large-sized species simply did not exist during the period. However, given that the majority of wasps known from Cretaceous amber are often of smaller proportions (i.e., 12 mm or less) (e.g., Liu et al. 2007; Engel and Grimaldi 2009; McKellar and Engel 2012; Engel et al. 2013, 2017), and this is also true for coeval ants (e.g., Engel and Grimaldi 2005; McKellar et al. 2013b, 2013c; Barden and Grimaldi 2013, 2014, 2016; Perrichot et al. 2016; Barden et al. 2017), one might conclude that the taphonomic bias is true. This is particularly evident when one considers that larger arthropod inclusions are certainly well known, with numerous such examples in these same resins (e.g., Grimaldi et al. 2002; Engel and Grimaldi 2008), and certainly this is the case in the diverse Cenozoic ambers (e.g., Engel 1995, 2014; Engel and Grimaldi 2007). Thus, the result of such a taphonomic bias would be that there are any number of ‘ghost’ lineages and for which there assuredly should have been representatives in the Cenomanian (e.g., larger ichneumonoids, varied sizeable aculeates [such as scoliids], siricoid wood wasps, &c.). However, given the adept flight of many Hymenoptera, a large-bodied wasp would have a better chance of avoiding contact with the flowing resin and, should it become entangled, would then have a similarly greater probability of freeing itself without damage. While this would certainly minimize the presence of Ichneumonidae, most of the primitive lineages of Braconidae are quite small, such as Rhyssalinae (Quicke 2015), and so should be more readily represented in amber. It is therefore quite vexing that braconids are, in fact, so rare in Cretaceous amber.

Parasitoid wasps of the family Braconidae remain a rarity in Cretaceous amber, despite the growing number of deposits with abundant arthropod inclusions (e.g., Azar et al. 2010; Perrichot and Néraudeau 2014; Grimaldi and Ross 2017). Nonetheless, the few species known, including *Seneciobracon novalatus* described herein, reveal a fauna largely composed of stem lineages, despite the fact that phylogenetic evidence would suggest crown-group representatives should also be present in at least the Upper Cretaceous. It is impossible to base broad-reaching conclusions on the overall composition of the Cretaceous braconid fauna and possible changes in composition through time with such an underwhelming amount of material at hand. This reality stresses the need for fieldwork to discover new fossil deposits and material from existing localities, so we might build a grander picture of parasitoid evolution during the Mesozoic.

## Supplementary Material

XML Treatment for Seneciobraconinae

XML Treatment for Seneciobracon

XML Treatment for Seneciobracon novalatus
